# Investigating Links Between Fear of COVID-19, Neuroticism, Social Networks Use Disorder, and Smartphone Use Disorder Tendencies

**DOI:** 10.3389/fpsyg.2021.682837

**Published:** 2021-08-02

**Authors:** Christian Montag, Cornelia Sindermann, Dmitri Rozgonjuk, Shixin Yang, Jon D. Elhai, Haibo Yang

**Affiliations:** ^1^Department of Molecular Psychology, Institute of Psychology and Education, Ulm University, Ulm, Germany; ^2^The Clinical Hospital of Chengdu Brain Science Institute, MOE Key Lab for Neuroinformation, University of Electronic Science and Technology of China, Chengdu, China; ^3^Institute of Mathematics and Statistics, University of Tartu, Tartu, Estonia; ^4^Academy of Psychology and Behavior, Faculty of Psychology, Tianjin Normal University, Tianjin, China; ^5^School of Psychology, Guizhou Normal University, Guiyang, China; ^6^Department of Psychology, and Department of Psychiatry, University of Toledo, Toledo, OH, United States

**Keywords:** COVID-19 pandemic, fear of COVID-19, social media addiction, smartphone addiction, Bergen social media addiction scale, smartphone addiction scale, neuroticism, personality

## Abstract

The present study investigates links between fear of COVID-19, the personality trait of neuroticism, social networks use disorder, and smartphone use disorder (SNUD and SmUD, respectively) tendencies. In an online survey, *N* = 932 participants recruited at a Chinese University (237 males and 695 females) completed self-reports on fear of COVID-19, neuroticism (and other personality traits from the Big Five Inventory-44), the Bergen Social Media Addiction Scale (assessing tendencies toward SNUD), and the Smartphone Addiction Scale short version—assessing individual differences in tendencies toward SmUD.

Our findings showed that all variables of main interest were positively correlated with each other. A mediation model suggested that SNUD (in parts) mediated the association between fear of COVID-19 and SmUD. Although neuroticism was robustly correlated with all mentioned variables, no moderation effect could be observed on the investigated fear-of-COVID-19-SNUD-link.

The findings of this work provide further evidence that the smartphone itself is only a device giving individuals access to software applications, which might be excessively used. Beyond that, the present data indicate neuroticism to be a risk factor with respect to fear of COVID-19, SNUD, and SmUD, although the study is limited by its cross-sectional study design.

## Introduction

The pandemic caused by coronavirus disease 2019 (COVID-19) has led to repeated lockdowns with social and physical distancing in 2020 across the world (Lau et al., 2020; Stiegler and Bouchard, 2020). Recent scientific literature has suggested that daily-life adversities related to problematic digital technology use have risen in 2020 (Király et al., 2020; Sun et al., 2020). A reason for this trend might be that social media use could represent a coping mechanism to being isolated and related negative affect (Kardefelt-Winther, 2014; Singh et al., 2020). Research from 2020 not only provides evidence for increases in Internet Use Disorders (IUD) in a broad sense (Sun et al., 2020), but it also highlights that, in particular, tendencies toward IUD in the realm of gaming and social media might have risen: a recent study showed tremendous rises in online time spent for gaming and social media use (DAK-Studie, 2020). Please note that a simple increase in online-time *per se* is not a sufficient predictor of problematic behavior in the realm of IUD, as (private) online time and tendencies toward IUD only correlate moderately (Sariyska et al., 2014). For a complex theoretical framework explaining the emergence and maintenance of IUD, please see Brand et al. (2016, 2019). In short, the authors proposed the I-PACE model. Here, a complex *I*nteraction of *P*erson, *A*ffect, *C*ognition, and *E*xecution variables is put forward to better understand IUDs.

The psychological construct “fear of COVID-19” has received a large amount of attention during the pandemic. A scale with the same name—the Fear of COVID-19 scale (we used the short version called FCV-19S; Ahorsu et al., 2020)—assesses the degree of being afraid of COVID-19. It encompasses physical symptoms (e.g., “heart is racing”) and sleep problems, among others. The scale itself has been associated not only with depressive (Zolotov et al., 2020) and other psychopathological symptoms (Martínez-Lorca et al., 2020), but also with the personality trait neuroticism (Caci et al., 2020). Moreover, earlier work from 2020 also showed associations between fear of COVID-19 and tendencies toward various IUDs. Positive associations between social networks use disorder (SNUD) and fear of COVID-19 have been demonstrated (Lin et al., 2020); and Elhai et al. (2020c) observed an association between COVID-19-anxiety and levels of smartphone use disorder (SmUD)^1^. SmUD can be seen as a mobile form of IUD (Montag et al., 2021). Moreover, it is important to note that problematic digital technology use, as observed with SmUD/SNUD, is robustly associated with neuroticism (Marengo et al., 2020a,b).

In summary, fear of COVID-19/COVID-19 anxiety^2^ seems to be associated with both SNUD and SmUD, and tendencies toward SmUD/SNUD may be a result of coping with COVID-19-related hardship. Robust associations with all variables and neuroticism have also been reported and, in general, neuroticism is well-known to be a risk factor for diverse (mental) health problems (Lahey, 2009). In terms of the already mentioned I-PACE model, the personality-variable of interest (neuroticism) to predict IUDs (SNUD/SmUD) belongs to the category of person variables (P-component of the model), whereas the fear of COVID-19 variable clearly functions as a stressor on the “Subjectively perceived situation” (p. 255) of a person, resulting in affective/cognitive responses (A- and C-components) and a decision to use an internet-related device (in our case the smartphone and/or social media/social networks [e.g. to cope with stress by using a digital application]).

With the present short report, we revisit the link between fear of COVID-19 and individual differences toward IUDs. In detail, we investigate fear of COVID-19 both in the context of SmUD and SNUD, while also considering neuroticism. As people often use their smartphones to access social media applications (SNUD/SmUD are strongly linked to each other; Sha et al., 2019; Rozgonjuk et al., 2020a,b), we expect that the association between fear of COVID-19 and SmUD is mediated by SNUD. The smartphone is only a vehicle—a medium—to access different applications and therefore we expect SNUD to be a mediator between fear of COVID-19 and SmUD in our work. To our knowledge, the variables personality, fear of COVID-19, SNUD, and SmUD have not been investigated before in a single study. Therefore, our work in part revisits associations such as between fear of COVID-19 and SNUD (see above), but it also goes beyond the published work as all mentioned variables are considered in a single study.

**Figure 1:**
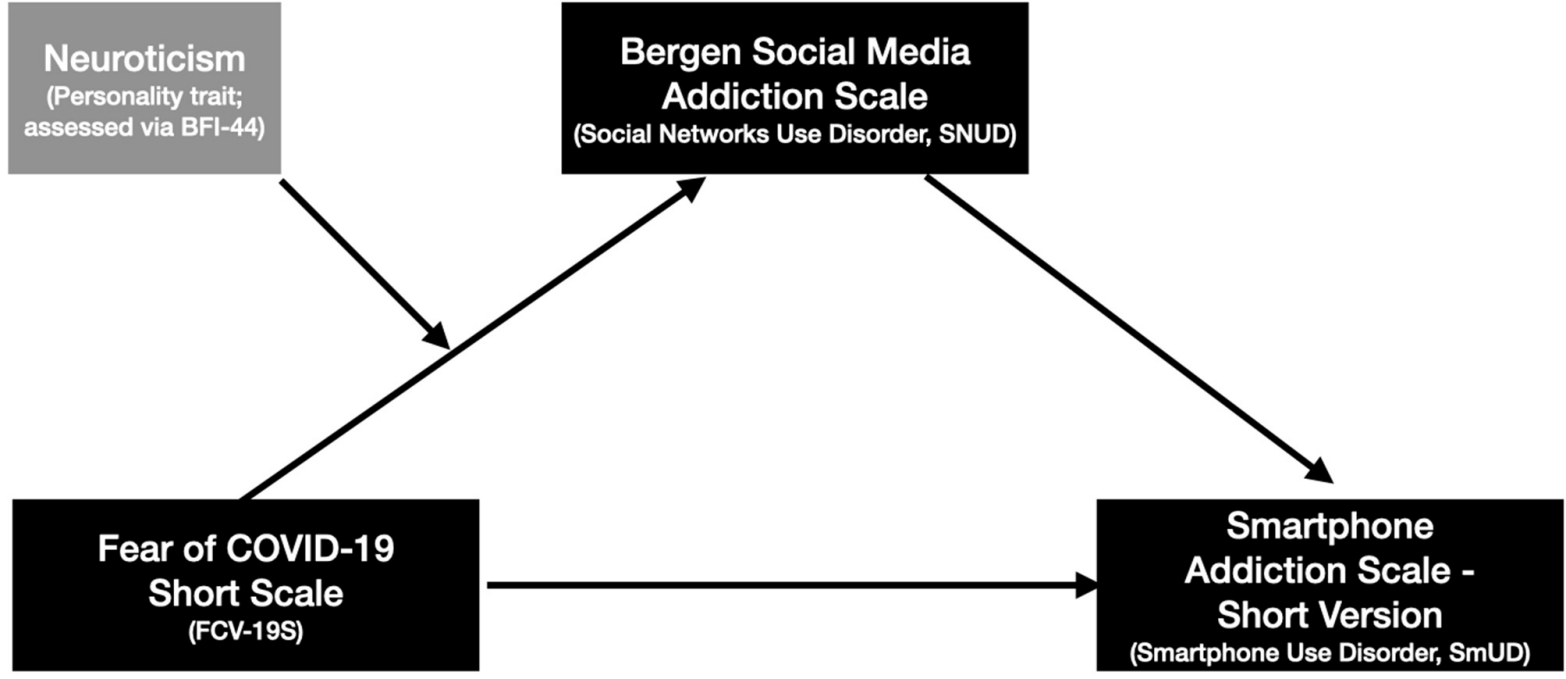
The hypothesized research model.

Since neuroticism is robustly linked with all constructs of relevance in the present work according to prior research, we investigated it as a moderator in the context of the mediation model (see also Figure 1). Many other models could have been proposed, but we decided to investigate neuroticism as a moderator (and not, for example, simply neuroticism as a predictor influencing “only” upon fear of COVID-19), because i) the literature has shown that neuroticism is associated with fear of COVID-19, SNUD, and SmUD and ii) empirical evidence highlights that neuroticism has been a moderator in other mental health associations (beyond the digital realm; Wasylkiw et al., 2010; Fadda and Scalas, 2016). We explicitly mention that this moderated mediation model is exploratory and has not been preregistered. All other associations mentioned here have been preregistered—please also note the detailed information in the method section.

Of note, we assessed individual differences of IUD in the present work via questionnaires developed against an addiction framework (Kwon et al., 2013; Bányai et al., 2017). We explicitly mention that both SNUD and SmUD are currently not officially diagnosable disorders and the current nomenclatures have been carefully chosen against a recent prominent theoretical framework (Brand et al., 2016), also considering the decision of the World Health Organization (WHO) to speak of Gaming Disorder (and not Gaming Addiction; Montag et al., 2019b; Pontes et al., 2019) in related addictive behaviors. More detailed discussion on this topic can be found in Montag et al. (2021) and Elhai et al. (2020a). In this context, we explicitly mention that we investigated individual differences in “tendencies” toward IUDs in the present work, as we largely relied on a student-based population. With the chosen nomenclature in the present work we also react to skepticism regarding the “addiction”-terminology in the realm of IUD (in particular SNUD/SmUD; Carbonell and Panova, 2017; Panova and Carbonell, 2018), and others currently might favor “problematic Internet use” which we also deem suitable. But as mentioned before, the here-presented terminology is in line with official labels for related recognized addictive behaviors such as gaming/gambling disorder.

To sum up, with the present work we aim to investigate whether the (positive) association between fear of COVID-19 and SmUD (assessed via the Smartphone Addiction Scale—short version) is mediated by SNUD (assessed via the Bergen Social Media Addiction Scale). On an exploratory note, we investigated the potential moderating impact of neuroticism on the associations between fear of COVID-19 and SNUD.

## Materials and Methods

### Participants

A total of *N* = 1,109 individuals were recruited in Tianjin, China. The survey was implemented on the platform https://www.wjx.cn/. Anyone from the age of 18 who understood simplified Chinese characters could participate. Nevertheless, due to the university background for the rollout of the study, mainly students participated (but they could also invite new study participants). Participants received monetary compensation for completing the survey: 8–12 Chinese RMB ($1.24-1.85 USD; 2021.05.03). The participants (including staff at the university) were enrolled via student courses or on the campus in 2020. All participants provided electronic informed consent and the study was approved by the local ethics committee.

Out of the initial sample of *N* = 1,109 participants, *n* = 91 people were excluded due to missing information (e.g., gender, personality, etc.). Moreover, we further excluded *n* = 8 participants who were underage and, therefore, not eligible for study participation (e.g., one person reported being 2 years old). Since we also assessed daily hours of smartphone use, we excluded all participants who reported > 16 hours each day on the smartphone (*n* = 23). This variable is not studied further, but presented in the descriptive statistics and has also been included for quality check reasons (higher smartphone usage should be associated with higher SmUD, but clearly only to a small extent, because online time alone is not sufficient to assess SmUD). Finally, people who indicated the same consecutive response option for more than 20 personality items (before re-coding reverse-coded items), indicating careless response patterns, were excluded (*n* = 55). The effective sample was *n* = 932 (237 men and 695 women; M_age_: 21.10, *SD* = 4.96 years). The distribution of highest education levels of the sample was as follows: Primary school graduation (0%), junior high school graduation (0,11%), graduated from high school (1,07%), studying for a bachelor's degree (92,70%), graduated from a bachelor's degree (4,83%), studying for a master's degree (0,54%), graduated from a master's degree (0,64%), doctoral study (0,11%), and doctoral degree (0%).

This study was preregistered at the Open Science Framework: https://osf.io/wk6be. As mentioned in the introduction, we expected in the preregistration the Bergen Social Media Addiction Scale, the Smartphone Addiction Scale (short version), and fear of COVID-19 to be positively associated with each other. Moreover, low conscientiousness and high neuroticism should be associated with both SNUD/SmUD and neuroticism should be positively associated with FCV-19S. Several more hypotheses have been put forward regarding the Need Satisfaction Inventory (NSI), but this could not be investigated (see below).

### Questionnaires

All participants filled in a Chinese version of the Bergen Social Media Addiction Scale [BSMAS; original by Andreassen et al. (2012), slightly modified using the social media term (instead of “Facebook”) and translated into Chinese with back and forward translation by the present group]. Moreover, participants completed a short form of the smartphone addiction scale (SAS-SV; Chinese version as used in Elhai et al., 2020b) and of the Fear of COVID-19 scale (FCV-19S; Ahorsu et al., 2020; Chi et al., 2021). Of importance, the SAS-SV and BSMAS instructions specifically asked participants about digital problems since the start of the COVID-pandemic. Finally, participants filled in the Chinese version of the Big Five Inventory (BFI-44; John et al., 1991; Pervin and John, 2003).

Please note that in the preregistration we also mentioned the Need Satisfaction Inventory (NSI, Lester, 1990). Data analysis revealed that the Chinese NSI version did not work properly from a psychometric perspective, at least in this sample. Therefore, the data of the NSI are not presented in this study.

The BSMAS consists of six items and was answered with a Likert scale ranging from 1–5. Higher scores indicate more severe problematic social media use or higher tendencies towards SNUD. The internal consistency estimate of the scale for the effective sample was Cronbach's α = 0.89. An exemplary item to be answered was: “Since the outbreak of the new coronavirus pneumonia epidemic, how many times have you used social media so much that it has had a negative impact on your job/studies?” In the original work by Andreassen et al. (2012) the BSMAS (with a focus on Facebook), among others, was correlated with neuroticism, extraversion (both positive), and conscientiousness (negative); there are comparable results in the present work with the exception of extraversion (see Table 1). We mention personality associations already here as a further validity check regarding the used Chinese questionnaire versions.

**Table 1:** Associations between the BFI-44 scales (personality traits in the left column), the BSMAS, the SAS-SV and the FCV-19S.

	**BSMAS**	**SAS-SV**	**FCV-19S**
Openness to experience	*r* = −0.006, *p* = 0.863	*r* = −0.066, *p* = 0.044	*r* = 0.017, *p* = 0.607
Conscientiousness	*r* = −0.146, *p* < 0.001	*r* = −0.272, *p* < 0.001	*r* = −0.040, *p* = 0.220
Extraversion	*r* = −0.005, *p* = 0.890	*r* = −0.106, *p* = 0.001	*r* = −0.025, *p* = 0.438
Agreeableness	*r* = −0.164, *p* < 0.001	*r* = −0.217, *p* < 0.001	*r* = −0.046, *p* = 0.156
Neuroticism	*r* = 0.219, *p* < 0.001	*r* = 0.316, *p* < 0.001	*r* = 0.186, *p* < 0.001

*FCV-19-S, Fear of COVID-19 short-scale; BSMAS, Bergen Social Media Addiction Scale; SAS-SV, Smartphone Addiction Scale—short version. Two-sided tests*.

The short version of the SAS-SV comprises 10 items and was answered with a Likert scale ranging from 1–6 reflecting on the time since the outbreak of COVID-19. An exemplary item to be answered was: “I have used my smartphone longer than intended.” Higher scores indicate higher tendencies toward SmUD. The long version of the SAS has been also correlated with the Big Five personality traits with strongest links to higher neuroticism and lower conscientiousness (Lachmann et al., 2019). Such a pattern between SmUD and personality has also recently been supported in a meta-analysis by Marengo et al. (2020b) and is in line with our findings in this study (see Table 1). The internal consistency estimate for the effective sample was Cronbach's α = 0.87.

The short version of the FCV-19 scale consists of seven items answered on a 1–5 Likert scale. The internal consistency estimate for FCV-19 was Cronbach's α = 0.89. An exemplary item was: “My heart races or palpitates when I think about getting coronavirus-19.” (see Ahorsu et al., 2020). Please note that higher scores in FCV-19S have been positively linked to neuroticism (as in the present study, see Table 1).

The BFI-44 responses ranged from 1–5 (strongly disagree to strongly agree), with higher scores indicating higher levels of a given trait. Importantly, some items were negatively scored and were therefore reverse-coded. We only used the neuroticism subscale in the current work for in depth-analysis, but see also the correlations presented in Table 1 featuring all Big Five personality traits. Neuroticism showed an internal consistency estimate of Cronbach's α = 0.72. An exemplary item for Neuroticism was: “I get nervous easily.” The remaining internal consistency estimates of personality traits were as follows: Extraversion = 0.69, Agreeableness = 0.67, Openness to Experience = 0.76, and Conscientiousness = 0.72.

### Statistical Analyses

The data were analyzed in SPSS and R. First, we calculated descriptive statistics and examined gender differences for reasons of simplicity in general by means of Welch two sample *t*-tests (given unequal variances for Conscientiousness and Agreeableness) and associations with age with Pearson correlations, respectively. Please note that similar results occurred with using *t*-tests for same variances. We mentioned in the preregistration that also education would be investigated as a nuisance variable, but given the homogenous sample (mostly students), we refrained from following this procedure. Next, we computed Pearson correlations to examine zero-order bivariate correlations between the main study variables (note that correlation results are not changing much with a Spearman correlation approach). We then computed a moderated mediation model where the outcome variable was SmUD, predicted from fear of COVID-19, and SNUD was treated as a mediating variable. Finally, neuroticism was treated as a moderator in the relationship between fear of COVID-19 and SNUD. We used the PROCESS Model 7 in R software. Specifically, we used the *model7()* function from the *processr* package (Markhwhiteii/Processr, n.d.). Confidence intervals and standard errors (SE) were bootstrapped over 5,000 samples.

## Results

### Descriptive Statistics, Gender Differences, and Associations With Age

Descriptive statistics are presented in Table 2. The descriptive statistics revealed that people on average reported medium scores regarding FCV-19S, the BSMAS, and SAS-SV, when taking into account the possible range of points on each scale. Self-reported smartphone usage averaged 6.38 h (*SD* = 2.96) each day. Please note that women on average showed higher scores compared to men regarding the FCV-19S (*M*_Females_ = 18.33 [*SD* = 6.03] vs. *M*_Males_ = 16.52 [*SD* = 6.30]) and self-reported daily hours spent on the smartphone (*M*_Females_ = 6.57 [*SD* = 2.96] vs. *M*_Males_ = 5.82 [*SD* = 2.91]). Regarding the Big Five personality traits, several gender differences occurred (see Table 2), but of particular interest in the present study are the higher neuroticism scores in females compared to males (*M*_Females_ = 3.09 [*SD* = 0.56] vs. *M*_Males_ = 2.81 [SD = 0.59]). For reasons of completeness, we also present descriptive statistics regarding all personality traits in Table 2. Age was not associated with any of the variables (with the exception of a small association with self-reported daily smartphone use in hours; see also Table 3).

**Table 2:** Descriptive statistics for the variables of interest in the total and in the male and female subsample separately.

**Variable names**	**Total sample (*n* = 932)**	**Men(*n* = 237)**	**Women (*n* = 695)**	**Welch two-sample *t*-tests regarding differences between males and females**
FCV-19S (possible range 7-35)	Mean = 17.87 (*SD* = 6.15)	Mean = 16.52 (*SD* = 6.30)	Mean = 18.33 (*SD* = 6.03)	*t*_(393.56)_ = −3.863, *p* < 0.001
BSMAS (possible range 6-30)	Mean = 16.42 (*SD* = 4.81)	Mean = 16.09 (*SD* = 4.87)	Mean = 16.53 (*SD* = 4.78)	*t*_(402.13)_ = −1.213, *p* = 0.226
SAS-SV (possible range 10-60)	Mean = 34.15 (*SD* = 9.40)	Mean = 33.15 (*SD* = 9.30)	Mean = 34.50 (*SD* = 9.41)	*t*_(412.59)_ = −1.925, *p* = 0.055
Self-reported daily hours on the smartphone(possible range 0-16 hours)	Mean = 6.38 (*SD* =2.96)	Mean = 5.82 (*SD* = 2.91)	Mean = 6.57 (*SD* = 2.96)	*t*_(414.09)_ = −3.390, *p* < 0.001
Openness to experience	Mean = 3.27 (*SD* = 0.52)	Mean = 3.37 (*SD* = 0.53)	Mean = 3.24 (*SD* = 0.51)	*t*_(398.19)_ = 3.312, *p* = 0.001
Conscientiousness	Mean = 3.11 (*SD* = 0.51)	Mean = 3.23 (*SD* = 0.57)	Mean = 3.07 (*SD* = 0.48)	*t*_(356.79)_ = 4.091, *p* < 0.001
Extraversion	Mean = 3.05 (*SD* = 0.55)	Mean = 3.19 (*SD* = 0.56)	Mean = 3.01 (*SD* = 0.54)	*t*_(395.57)_ = 4.459, *p* < 0.001
Agreeableness	Mean = 3.69 (*SD* = 0.48)	Mean = 3.72 (*SD* = 0.53)	Mean = 3.69 (*SD* = 0.46)	*t*_(364.72)_ = 0.948, *p* = 0.344
Neuroticism	Mean = 3.02 (*SD* = 0.58)	Mean = 2.81 (*SD* = 0.59)	Mean = 3.09 (*SD* = 0.56)	*t*_(391.91)_ = −6.207, *p* < 0.001

*FCV-19S, Fear of COVID-19 Short-Scale; BSMAS, Bergen Social Media Addiction Scale; SAS-SV, Smartphone Addiction Scale—Short Version*.

**Table 3:** Pearson correlations between all metric variables of interest.

	**BSMAS**	**SAS-SV**	**Self-reported smartphone use in hours per days**	**Age**
FCV-19S	*r* = 0.321, *p* < 0.001	*r* = 0.316, *p* < 0.001	*r* = −0.017, *p* = 0.609	*r* = 0.022, *p* = 0.511
BSMAS		*r* = 0.630, *p* < 0.001	*r* = 0.065, *p* = 0.047	*r* = −0.058, *p* = 0.076
SAS-SV			*r* = 0.149, *p* < 0.001	*r* = −0.013, *p* = 0.687
Self-reported smartphone use in hours per day				*r* = −0.129, *p* < 0.001

*FCV-19S, Fear of COVID-19 Short-Scale; BSMAS, Bergen Social Media Addiction Scale; SAS-SV, Smartphone Addiction Scale—Short Version. Two-sided tests*.

### Associations Between the Fear of COVID-19S Scale, the Bergen Social Media Addiction Scale, and the Smartphone Addiction Scale-Short Version

Table 3 depicts correlations between the metric variables FCV-19S, BSMAS, SAS-SV, and smartphone use in hours and age. As one can see, FCV-19S, BSMAS, and SAS-SV were robustly and positively associated with each other. Whereas FCV-19S and the problematic digital technology use variables are moderately associated with each other (BSMAS: *r* = 0.321, *p* < 0.001; SAS-SV: *r* = 0.316, *p* < 0.001), the SAS-SV and BSMAS showed high associations with each other (*r* = 0.630, *p* < 0.001). Self-reported smartphone use in hours per day was positively associated with the SAS-SV (as expected), but associations were not overly large (*r* = 0.149, *p* < 0.001), lending support to the idea that self-reported usage-hours *per se* are not a sufficient predictor of use disorder tendencies (Sariyska et al., 2014). As mentioned, age associations were negligible.

### Associations of the Big Five Personality Traits With the Fear of COVID-19S Scale, the Bergen Social Media Addiction Scale, and the Smartphone Addiction Scale-Short Version

The typical associations between the Big Five Personality traits assessed with the BFI and SmUD assessed with SAS-SV could be observed (see Table 1). As hypothesized in the preregistration, lower conscientiousness scores / higher neuroticism scores went along with higher SAS-SV scores (*r* = −0.272, *p* < 0.001 / *r* = 0.316, *p* < 0.001). These findings could also be extended to BSMAS scores here (this was also a preregistered hypothesis; *r* = −0.146, *p* < 0.001 / *r* = 0.219, *p* < 0.001). Moreover, as reported in the literature (Caci et al., 2020; Lee and Crunk, 2020) and preregistered results, higher FCV-19S scores exclusively (and significantly) linked to higher scores in neuroticism (*r* = 0.186, *p* < 0.001).

### Moderated Mediation Model

The moderated mediation model depicted in Table 4 shows there were significant direct effects of FCV-19S (a1), as well as neuroticism on BSMAS (a2). Yet, the moderating effect of neuroticism in the relationship between FCV-19S and SNUD (BSMAS) was not statistically significant (a3). This said, the direct effect of SNUD (BSMAS) on SmUD (SAS-SV; b), as well as the effect of FCV-19S on SmUD (SAS-SV), after controlling for SNUD (BSMAS), were significant (c'). Since the index of moderated mediation was not statistically significant (judged by confidence intervals in IMM), this implies that there was no moderating mediation effect in the model. This may be the case, because levels of neuroticism do not introduce differences in mediating effects. Finally, the mediating effect of SNUD (BSMAS) in the relationship between FCV-19S and SmUD (SAS-SV) was present, as indicated by the coefficients of indirect effects (when the effects of neuroticism are accounted for) and judged by the confidence intervals (c1-c3). In other words, the indirect effect of SNUD (BSMAS) on the relationship between FCV-19S and SAS-SV was positive and significant on the −1 SD, average, and +1 SD levels of neuroticism.

**Table 4:** Moderated mediation model results.

**Path**	**Coefficient**	**SE**	**z**	***p*-value**	**Lower CI**	**Upper CI**
a1: Direct effect of FCV-19S on BSMAS	0.352	0.144	2.439	0.015	0.071	0.636
a2: Direct effect of Neuroticism on BSMAS	2.052	0.858	2.393	0.017	0.340	3.755
a3: The interaction effect (Neuroticism x FCV-19S) on BSMAS	-0.042	0.047	−0.891	0.373	−0.133	0.051
b: Direct effect of BSMAS on SAS-SV	1.151	0.059	19.627	0.000	1.028	1.262
c': The effect of FCV-19S on SAS-SV, after controlling for BSMAS	0.195	0.045	4.303	0.000	0.108	0.285
IMM	-0.048				−0.156	0.058
c1: Ind (low Neuroticism)	0.288				0.198	0.385
c2: Ind (medium Neuroticism)	0.260				0.197	0.331
c3: Ind (high Neuroticism)	0.232				0.145	0.323

*a1, direct effect of the independent variable on the mediator; a2, direct effect of the moderator variable on the mediator; a3, the interaction effect of the moderator and the independent variable on mediator; b, path from the mediator to dependent variable; c', direct effect of the independent variable on the dependent variable after controlling for the mediator; IMM, index of moderated mediation (tests if moderated mediation is present); Ind (low), Ind (medium), Ind (high), indirect effects of the mediator on different levels of the moderator (−1 SD, average, and +1 SD). Further abbreviations: FCV-19S, Fear of COVID-19 Short Scale; BSMAS, Bergen Social Media Addiction Scale; SAS-SV, Smartphone Addiction Scale—Short Version*.

## Discussion

As expected in our preregistration, we observed positive associations between fear of COVID-19 and both SNUD/SmUD. We also observed a positive association between fear of COVID-19 and neuroticism, as expected and preregistered. The same was true for the neuroticism-SNUD/SmUD associations (and the low conscientiousness—higher SNUD/SmUD associations). These results underline that neuroticism represents a risk factor not only for experiencing fear of COVID-19, but also for greater tendencies toward SmUD and SNUD. Please note that we only had correlational data and therefore the present results should be interpreted cautiously with regards to causality. Yet, since neuroticism is relatively stable over time (McCrae and Costa, 2003), it is plausible that neuroticism impacts other variables (and not the other way around).

Beyond our preregistration, we hypothesized that SNUD might mediate relations between fear of COVID-19 and SmUD. This is indeed what we, in part, observed. The mediation model showed that there was an indirect effect of fear of COVID-19 on SmUD via SNUD, but after controlling for the mediator, a direct effect from fear of COVID-19 on SmUD was still visible.

Given the existence of the indirect effect, our findings support the idea that the smartphone as a device is just a vehicle to access certain software applications, and likely not causing the problem itself (Lowe-Calverley and Pontes, 2020). Mounting evidence in the literature—hinting toward social media/messenger applications as important drivers of SmUD [see also the works by Sha et al. (2019) and Rozgonjuk et al. (2020a,b)]—show the relevance to more strongly focus future research on how the smartphone is used (both in terms of usage intensity and choice of applications) instead of focusing only on broad (excessive) smartphone use. Such a narrower research focus would also have consequences regarding interventions on how to better reduce excessive use of the smartphone. If it appears that, for instance, social media applications are among the main channels driving excessive use of the smartphone, interventions should aim at reducing the use of these applications, but likely not applications such as the map service, to name another prominent feature of the smartphone helping humans to navigate in unknown territory. Of note, such interventions could not only target individual social media/smartphone users, but also the regulation of social media platforms. In recent works by Montag et al. (2019a) and Montag and Hegelich (2020) it has been explained in detail how the data business model behind social media services led to the design of “addictive” social media apps luring in users as often as possible to spend a maximum of time on the platforms, reacting to as many posts as possible (this means even more data to be exploited by the tech-companies). Rethinking the data business model behind many social media services could result in “healthier” social media platforms with lower addictive potential (Sindermann et al., 2020). Moreover, we note that psychological processes such as Fear of Missing Out (FoMO) can be triggered by platform design (Alutaybi et al., 2019), and FoMO itself is linked to social media (and smartphone) use disorder tendencies (Elhai et al., 2020d), likely amplified by the COVID-19 crisis. During the pandemic, people need to inform themselves on a daily basis with respect to the current state of affairs regarding the infection rate in one's own community, and social media represents one channel to obtain information (although there have been disinformation campaigns causing problems; Sharma et al., 2020; Tasnim et al., 2020). Thus, greater fear of COVID-19 could have resulted in higher SNUD, perhaps not only due to constant monitoring of the COVID-19 situation on social media, but also to distract oneself with different content from daily negative affect due to the pandemic and associated social isolation (as also mentioned in the introduction). In this context, it is also imaginable that associations such as between fear of COVID-19 and SNUD would be moderated by neuroticism. However, although positive associations between neuroticism and all other focus variables could be observed as preregistered, such a moderating effect was not the case—at least not as hypothesized and depicted in Figure 1. Hence the association between fear of COVID-19 and SNUD did not differ depending on neuroticism levels.

In sum, the present work replicates earlier findings that fear of COVID-19 is linked to both SNUD and SmUD, but also demonstrates that SNUD is in part a mediator between fear of COVID-19 and SmUD. Bivariate associations with personality are as expected. The interpretation of the results of our study is limited by the correlational nature of the collected data. For instance, it is also imaginable that SNUD results in higher fear of COVID-19, given that individuals are constantly confronted on social media channels with many negative reports about the pandemic. We also mention that our sample consisted mainly of students, therefore it is of importance to carry out further work with participants from different sociodemographic backgrounds. Finally, we mention that it is of importance to replicate these findings in other cultural settings, as the present findings stem from a Chinese sample.

## Data Availability Statement

The original contributions (aggregated data) presented in the study are included in the article/Supplementary Material, further inquiries can be directed to the corresponding author/s.

## Ethics Statement

The studies involving human participants were reviewed and approved by the ethics committee at Tianjin Normal University. The ethics committee waived the requirement of written informed consent for participation.

## Author Contributions

CM and CS designed the present study. CM wrote the first draft of the manuscript including running statistical analysis. DR supported the statistical analysis regarding the moderated mediation model. CS, SY and HY conducted the back and forth-translations for the measures not being available in Chinese language. SY and HY carried out the data collection in Tianjin. JDE critically revised the paper. Finally, all authors worked over the final draft again and approved the last version of this paper.

## Conflict of Interest

The authors declare that the research was conducted in the absence of any commercial or financial relationships that could be construed as a potential conflict of interest.

## Publisher's Note

All claims expressed in this article are solely those of the authors and do not necessarily represent those of their affiliated organizations, or those of the publisher, the editors and the reviewers. Any product that may be evaluated in this article, or claim that may be made by its manufacturer, is not guaranteed or endorsed by the publisher.

## Footnotes

^1^Although they observed that controlling for depression/anxiety resulted in COVID-19 anxiety no longer predicting problematic smartphone use (PSU; as the authors call it in their work).

^2^Please note that theories such as the revised reinforcement sensitivity theory distinguish between the emotions of anxiety and fear (Reuter et al., 2015). Given the brevity of this article, we do not explain this further here.

## References

[B1] Ahorsu D. K. Lin C.-Y. Imani V. Saffari M. Griffiths M. D. Pakpour A. H. (2020). The fear of COVID-19 scale: development and initial validation. Int. J. Ment. Health Addict. 1–9. 10.1007/s11469-020-00270-8 32226353 PMC7100496

[B2] Alutaybi A. Arden-Close E. McAlaney J. Stefanidis A. Phalp K. Ali R. (2019). How can social networks design trigger fear of missing out?, in 2019 IEEE International Conference on Systems, Man and Cybernetics (Bari: SMC), 3758–3765. 10.1109/SMC.2019.8914672

[B3] Andreassen C. S. Torsheim T. Brunborg G. S. Pallesen S. (2012). Development of a Facebook addiction scale. Psychol. Rep. 110, 501–517. 10.2466/02.09.18.PR0.110.2.501-517 22662404

[B4] Bányai F. Zsila Á. Király O. Maraz A. Elekes Z. Griffiths M. D.. (2017). Problematic social media use: results from a large-scale nationally representative adolescent sample. PLoS ONE 12:e0169839. 10.1371/journal.pone.0169839 28068404 PMC5222338

[B5] Brand M. Wegmann E. Stark R. Müller A. Wölfling K. Robbins T. W.. (2019). The Interaction of Person-Affect-Cognition-Execution (I-PACE) model for addictive behaviors: update, generalization to addictive behaviors beyond internet-use disorders, and specification of the process character of addictive behaviors. Neurosci. Biobehav. Rev. 104, 1–10. 10.1016/j.neubiorev.2019.06.032 31247240

[B6] Brand M. Young K. S. Laier C. Wölfling K. Potenza M. N. (2016). Integrating psychological and neurobiological considerations regarding the development and maintenance of specific Internet-use disorders: an Interaction of Person-Affect-Cognition-Execution (I-PACE) model. Neurosci. Biobehav. Rev. 71, 252–266. 10.1016/j.neubiorev.2016.08.033 27590829

[B7] Caci B. Miceli S. Scrima F. Cardaci M. (2020). Neuroticism and fear of COVID-19. The interplay between boredom, fantasy engagement, and perceived control over time. Front. Psychol. 11:574393. 10.3389/fpsyg.2020.574393 33154730 PMC7588354

[B8] Carbonell X. Panova T. (2017). A critical consideration of social networking sites' addiction potential. Addict. Res. Theory 25, 48–57. 10.1080/16066359.2016.1197915

[B9] Chi X. Chen S. Chen Y. Chen D. Yu Q. Guo T.. (2021). Psychometric evaluation of the fear of COVID-19 scale among Chinese population. Int. J. Ment. Health Addict. 1–16. 10.1007/s11469-020-00441-7 33456407 PMC7799163

[B10] DAK-Studie (2020). DAK-Studie: Gaming, Social-Media & Corona. Available online at: https://www.dak.de/dak/gesundheit/dak-studie-gaming-social-media-und-corona-2295548.html (accessed June 30, 2021).

[B11] Elhai J. D. Yang H. Fang J. Bai X. Hall B. J. (2020b). Depression and anxiety symptoms are related to problematic smartphone use severity in Chinese young adults: fear of missing out as a mediator. Addict. Behav. 101:105962. 10.1016/j.addbeh.2019.04.020 31030950

[B12] Elhai J. D. Yang H. Levine J. C. (2020a). Applying fairness in labeling various types of internet use disorders: commentary on how to overcome taxonomical problems in the study of internet use disorders and what to do with “smartphone addiction”? J. Behav. Addict. 1, 924–927. 10.1556/2006.2020.00071 PMC8969732 33027061

[B13] Elhai J. D. Yang H. McKay D. Asmundson G. J. G. (2020c). COVID-19 anxiety symptoms associated with problematic smartphone use severity in Chinese adults. J. Affect. Disord. 274, 576–582. 10.1016/j.jad.2020.05.080 32663990 PMC7251360

[B14] Elhai J. D. Yang H. Montag C. Elhai J. D. Yang H. Montag C. (2020d). Fear of missing out (FOMO): overview, theoretical underpinnings, and literature review on relations with severity of negative affectivity and problematic technology use. Braz. J. Psychiatry.43, 203–209. 10.1590/1516-4446-2020-0870 32401865 PMC8023172

[B15] Fadda D. Scalas L. F. (2016). Neuroticism as a moderator of direct and mediated relationships between introversion-extraversion and well-being. Eur. J. Psychol. 12, 49–67. 10.5964/ejop.v12i1.985 27247693 PMC4873067

[B16] John O. P. Donahue E. M. Kentle R. L. (1991). Big Five Inventory. Washington, DC: APA PsycTests. 10.1037/t07550-000

[B17] Kardefelt-Winther D. (2014). A conceptual and methodological critique of internet addiction research: Towards a model of compensatory internet use. Comput. Hum. Behav. 31, 351–354. 10.1016/j.chb.2013.10.059

[B18] Király O. Potenza M. N. Stein D. J. King D. L. Hodgins D. C. Saunders J. B.. (2020). Preventing problematic internet use during the COVID-19 pandemic: consensus guidance. Compr. Psychiatry 100:152180. 10.1016/j.comppsych.2020.152180 32422427 PMC7215166

[B19] Kwon M. Kim D.-J. Cho H. Yang S. (2013). The smartphone addiction scale: development and validation of a short version for adolescents. PLoS ONE 8:e83558. 10.1371/journal.pone.0083558 24391787 PMC3877074

[B20] Lachmann B. Duke É. Sariyska R. Montag C. (2019). Who's addicted to the smartphone and/or the Internet? Psychol. Pop. Media Cult. 8, 182–189. 10.1037/ppm0000172

[B21] Lahey B. B. (2009). Public health significance of neuroticism. Am. Psychol. 64, 241–256. 10.1037/a0015309 19449983 PMC2792076

[B22] Lau H. Khosrawipour V. Kocbach P. Mikolajczyk A. Schubert J. Bania J.. (2020). The positive impact of lockdown in Wuhan on containing the COVID-19 outbreak in China. J. Travel Med. 27:taaa037. 10.1093/jtm/taaa037 32181488 PMC7184469

[B23] Lee S. A. Crunk E. A. (2020). Fear and psychopathology during the COVID-19 crisis: neuroticism, hypochondriasis, reassurance-seeking, and coronaphobia as fear factors. OMEGA J. Death Dying. 10.1177/0030222820949350 32762291

[B24] Lester D. (1990). Maslow's hierarchy of needs and personality. Pers. Individ. Dif. 11, 1187–1188. 10.1016/0191-8869(90)90032-M

[B25] Lin C.-Y. Broström A. Griffiths M. D. Pakpour A. H. (2020). Investigating mediated effects of fear of COVID-19 and COVID-19 misunderstanding in the association between problematic social media use, psychological distress, and insomnia. Internet Intervent. 21:100345. 10.1016/j.invent.2020.100345 32868992 PMC7449889

[B26] Lowe-Calverley E. Pontes H. M. (2020). Challenging the concept of smartphone addiction: an empirical pilot study of smartphone usage patterns and psychological well-being. Cyberpsychol. Behav. Soc. Netw. 23, 550–556. 10.1089/cyber.2019.0719 32498607

[B27] Marengo D. Poletti I. Settanni M. (2020a). The interplay between neuroticism, extraversion, and social media addiction in young adult Facebook users: testing the mediating role of online activity using objective data. Addict. Behav. 102:106150. 10.1016/j.addbeh.2019.106150 31706139

[B28] Marengo D. Sindermann C. Häckel D. Settanni M. Elhai J. D. Montag C. (2020b). The association between the Big Five personality traits and smartphone use disorder: a meta-analysis. J. Behav. Addict. 9, 534–550. 10.1556/2006.2020.00069 33011714 PMC8943667

[B29] Markhwhiteii/Processr. (n.d.). README.md. Retrieved from https://rdrr.io/github/markhwhiteii/processr/f/README.md (accessed December 20, 2020).

[B30] Martínez-Lorca M. Martínez-Lorca A. Criado-Álvarez J. J. Armesilla M. D. C. Latorre J. M. (2020). The fear of COVID-19 scale: validation in spanish University students. Psychiatry Res. 293:113350. 10.1016/j.psychres.2020.113350 32777619 PMC7396130

[B31] McCrae R. R. Costa P. T. (2003). Personality in adulthood: a five-factor theory perspective. New York, NY: Guilford Press. 10.4324/9780203428412

[B32] Montag C. Hegelich S. (2020). Understanding detrimental aspects of social media use: will the real culprits please stand up? Front. Sociol. 5:599270. 10.3389/fsoc.2020.599270 33869524 PMC8022744

[B33] Montag C. Lachmann B. Herrlich M. Zweig K. (2019a). Addictive features of social media/messenger platforms and freemium games against the background of psychological and economic theories. Int. J. Environ. Res. Public Health 16:2612. 10.3390/ijerph16142612 31340426 PMC6679162

[B34] Montag C. Schivinski B. Sariyska R. Kannen C. Demetrovics Z. Pontes H. M. (2019b). Psychopathological symptoms and gaming motives in disordered gaming—a psychometric comparison between the WHO and APA diagnostic frameworks. J.Clin. Med. 8:1691. 10.3390/jcm8101691 31618950 PMC6832511

[B35] Montag C. Wegmann E. Sariyska R. Demetrovics Z. Brand M. (2021). How to overcome taxonomical problems in the study of Internet use disorders and what to do with “smartphone addiction”?. J. Behav. Addict. 9, 908–914. 10.1556/2006.8.2019.59 31668089 PMC8969715

[B36] Panova T. Carbonell X. (2018). Is smartphone addiction really an addiction? J. Behav. Addict. 7, 252–259. 10.1556/2006.7.2018.49 29895183 PMC6174603

[B37] Pervin L. A. John O. P. (2003). Personality: Theory and Research (Chinese Version). Shanghai: East China Normal University Press.

[B38] Pontes H. M. Schivinski B. Sindermann C. Li M. Becker B. Zhou M.. (2019). Measurement and conceptualization of gaming disorder according to the world health organization framework: the development of the gaming disorder test. Int. J. Ment. Health Addict. 19, 508–528. 10.1007/s11469-019-00088-z

[B39] Reuter M. Cooper A. J. Smillie L. D. Markett S. Montag C. (2015). A new measure for the revised reinforcement sensitivity theory: psychometric criteria and genetic validation. Front. Syst. Neurosci. 9:38. 10.3389/fnsys.2015.00038 25852497 PMC4360558

[B40] Rozgonjuk D. Sindermann C. Elhai J. D. Christensen A. P. Montag C. (2020b). Associations between symptoms of problematic smartphone, Facebook, WhatsApp, and Instagram use: an item-level exploratory graph analysis perspective. J. Behav. Addict. 9, 686–697. 10.1556/2006.2020.00036 32986606 PMC8943679

[B41] Rozgonjuk D. Sindermann C. Elhai J. D. Montag C. (2020a). Comparing smartphone, WhatsApp, Facebook, Instagram, and Snapchat: which platform elicits the greatest use disorder symptoms? Cyberpsychol. Behav. Soc. Netw. 24, 129–134. 10.1089/cyber.2020.0156 32907403

[B42] Sariyska R. Reuter M. Bey K. Sha P. Li M. Chen Y.-F.. (2014). Self-esteem, personality and Internet addiction: a cross-cultural comparison study. Pers. Individ. Dif. 61–62, 28–33. 10.1016/j.paid.2014.01.001

[B43] Sha P. Sariyska R. Riedl R. Lachmann B. Montag C. (2019). Linking internet communication and smartphone use disorder by taking a closer look at the Facebook and WhatsApp applications. Addict. Behav. Rep. 9:100148. 10.1016/j.abrep.2018.100148 31193857 PMC6543448

[B44] Sharma K. Seo S. Meng C. Rambhatla S. Liu Y. (2020). COVID-19 on Social Media: Analyzing Misinformation in Twitter Conversations. arxiv[Preprint].arxiv:2003.12309. Available online at: http://arxiv.org/abs/2003.12309 (accessed October 22, 2020).

[B45] Sindermann C. Kuss D. J. Throuvala M. A. Griffiths M. D. Montag C. (2020). Should we pay for our social media/messenger applications? preliminary data on the acceptance of an alternative to the current prevailing data business model. Frontiers in Psychology 11:1415. 10.3389/fpsyg.2020.01415 32760312 PMC7371851

[B46] Singh S. Dixit A. Joshi G. (2020). Is compulsive social media use amid COVID-19 pandemic addictive behavior or coping mechanism? Asian J. Psychiatry 54:102290. 10.1016/j.ajp.2020.102290 PMC7338858 32659658

[B47] Stiegler N. Bouchard J.-P. (2020). South Africa: challenges and successes of the COVID-19 lockdown. Annales Médico-Psychologiques Revue Psychiatrique 178, 695–698. 10.1016/j.amp.2020.05.006 32836300 PMC7250766

[B48] Sun Y. Li Y. Bao Y. Meng S. Sun Y. Schumann G.. (2020). Brief report: increased addictive internet and substance use behavior during the COVID-19 pandemic in China. Am. J. Addict. 29, 268–270. 10.1111/ajad.13066 32500608 PMC7300868

[B49] Tasnim S. Hossain M. M. Mazumder H. (2020). Impact of rumors and misinformation on COVID-19 in social media. J. Prevent. Med. Public Health 53, 171–174. 10.3961/jpmph.20.094 32498140 PMC7280809

[B50] Wasylkiw L. Fabrigar L. R. Rainboth S. Reid A. Steen C. (2010). Neuroticism and the architecture of the self: exploring neuroticism as a moderator of the impact of ideal self-discrepancies on emotion. J. Pers. 78, 471–492. 10.1111/j.1467-6494.2010.00623.x 20433627

[B51] Zolotov Y. Reznik A. Bender S. Isralowitz R. (2020). COVID-19 fear, mental health, and substance use among Israeli University students. Int. J. Ment. Health Addict. 1–7. 10.1007/s11469-020-00351-8 32837432 PMC7299139

